# Detection of TRPM6 and TRPM7 Proteins in Normal and Diseased Cardiac Atrial Tissue and Isolated Cardiomyocytes

**DOI:** 10.3390/ijms232314860

**Published:** 2022-11-28

**Authors:** Inga Andriulė, Dalia Pangonytė, Asfree Gwanyanya, Dainius Karčiauskas, Kanigula Mubagwa, Regina Mačianskienė

**Affiliations:** 1 Institute of Cardiology, Lithuanian University of Health Sciences, 50103 Kaunas, Lithuania; 2 Department of Human Biology, Faculty of Health Sciences, University of Cape Town, Cape Town 7700, South Africa; 3 Department of Cardiac, Thoracic and Vascular Surgery, Hospital of Lithuanian University of Health Sciences Kauno Klinikos, 50161 Kaunas, Lithuania; 4 Department of Cardiovascular Sciences, Faculty of Medicine, KU Leuven, 3000 Leuven, Belgium; 5 Department of Basic Sciences, Faculty of Medicine, Université Catholique de Bukavu, Bukavu, Congo

**Keywords:** TRPM6 and TRPM7 channels, atrial myocyte and tissue, ischemic heart disease, atrial fibrillation

## Abstract

Magnesium-sensitive transient receptor potential melastatin (TRPM) ion channels, TRPM6 and TRPM7, are present in several organs, but their roles in the heart remain unclear. Therefore, here, we studied the expression patterns of TRPM6 and TRPM7 in normal and diseased myocardium. Cardiac atrial tissue and cardiomyocytes were obtained from healthy pigs and undiseased human hearts as well as from hearts of patients with ischemic heart disease (IHD) or atrial fibrillation (AF). Immunofluorescence and ELISA were used to detect TRP proteins. TRPM6 and TRPM7 immunofluorescence signals, localized at/near the cell surface or intracellularly, were detected in pig and human atrial tissues. The TRP channel modulators carvacrol (CAR, 100 µM) or 2-aminoethoxydiphenyl borate (2-APB, 500 µM) decreased the TRPM7 signal, but enhanced that of TRPM6. At a higher concentration (2 mM), 2-APB enhanced the signals of both proteins. TRPM6 and TRPM7 immunofluorescence signals and protein concentrations were increased in atrial cells and tissues from IHD or AF patients. TRPM6 and TRPM7 proteins were both detected in cardiac atrial tissue, with relatively similar subcellular localization, but distinctive drug sensitivity profiles. Their upregulated expression in IHD and AF suggests a possible role of the channels in cardiac atrial disease.

## 1. Introduction

Until now, the cardiac expressions and functional roles of TRPM7 and TRPM6 ion channels, two magnesium-sensitive members of the transient receptor potential (TRP) melastatin subfamily of channels, have remained unclear. Electrophysiological studies have documented the presence of channels that are regulated by intracellular magnesium in cardiac myocytes [1,2,3,4,5,6] (for review see [7]) and in other cells of the heart, mainly in fibroblasts [8,9] (for review see [10]) and in pacemaker cells [11] (for review see [12]), suggesting the expression of TRPM7 and/or TRPM6 in the heart. In various other tissues, these TRPM channels have been proposed to mediate divalent cation homeostasis (for review see [13]), however, although a similar role in Mg^2+^ homeostasis has been proposed [14], their broader role in the heart remains to be uncertain. Furthermore, these channels have been shown to be upregulated in cardiac disease conditions such as atrial fibrillation (AF) [8,15], however, the implications in cardiovascular pathophysiology still require further clarifications.

The known ubiquitous expression of TRPM7 in all mammalian cells [16,17] and the relative restriction of TRPM6 to only some tissues (such as the tubular epithelium of the kidney, placenta, and intestine) [18,19] have led to the suggestion that only TRPM7 is responsible for magnesium-sensitive channels in the heart. This concept has been supported by several studies in which the expression of TRPM6 could not be demonstrated. However, some studies have identified *trpm6* mRNA and protein in the human heart and suggested an increase in right atria (RA) during AF [15]. Among such studies, our previous findings indicated the presence of TRPM6 in all chamber walls of the human heart [20], but the subject has remained controversial due to questions about the specificity of immunological tools used to detect the protein.

The aim of the present study was to further determine the expression of TRPM6 and TRPM7 channel proteins in porcine and human cardiac myocytes and tissues, using more specific detection methods, and to obtain information on their subcellular localization. In addition, we wanted to further evaluate the effect of treating cells with TRP channel modulators as well as of cardiac pathology on the detected channel expression.

## 2. Results

### 2.1. TRP Detection in Isolated Single Cardiomyocytes

**Figure 1:**
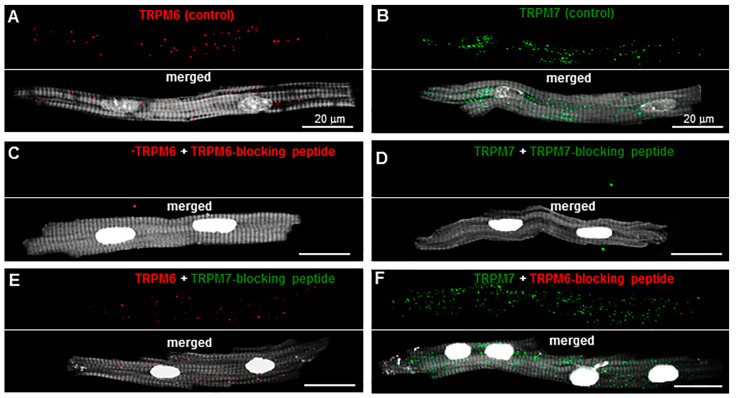
Specificity of antibody-based detection of TRPM6 and TPRM7 in pig right atrial (RA) cardiomyocytes: (**A**,**B**) Immunofluorescence signals in cells incubated with anti-TRPM6 (red, when using Alexa Fluor 546) or anti-TRPM7 (green, when using Alexa Fluor 488) antibodies used alone (upper panels). The immunofluorescence of TRPM6 was increased post-acquisition by a factor of 2 for better visualization of the red staining. Bottom panels show merged images in the same cells stained with either anti-TRPM6 or anti-TRPM7 as well as Phalloidin-CF 405 for filamentous actin (F-actin) cytoskeleton (in surrogate grey) and Hoechst 33342 for nuclei (in surrogate white); (**C**,**D**) immunofluorescence signals in cells incubated with anti-TRPM6 or anti-TRPM7 antibodies in the presence of antibody-blocking peptides (upper panels). Bottom panels show merged images in the same cells stained with either anti-TRPM6 or anti-TRPM7, together with the blocking peptides, as well as Phalloidin-CF 405 for F-actin cytoskeleton (in surrogate grey) and Hoechst 33342 for nuclei (in surrogate white). There was no staining with anti-TRPM6 (red) or anti-TRPM7 (green) in the presence of antibody-blocking peptides; (**E**,**F**) immunofluorescence signals in cells incubated with anti-TRPM6 antibodies in the presence of the TRPM7-blocking peptide or with anti-TRPM7 antibodies in the presence of the TRPM6-blocking peptide (upper panels). Bottom panels show merged images in the same cells stained with the anti-TRPM antibodies together with the off-target blocking peptides, as well as Phalloidin-CF 405 for F-actin cytoskeleton (in surrogate grey) and Hoechst 33342 for nuclei (in surrogate white). Staining was similar to that in conditions where there is no blocking peptide. Scale bars indicate 20 µm.

**Figure 2:**
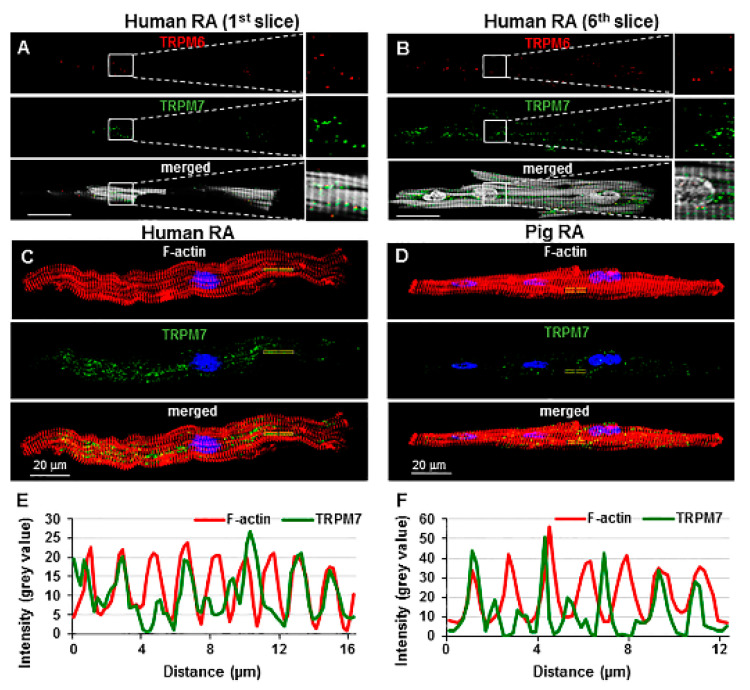
Detection of TRPM6 and TRPM7 in the same cell and cellular localization of TRPM7: (**A**,**B**) Images of human RA cell illustrating the localization of TRPM6 (red) and TRPM7 (green) proteins in the same cardiomyocyte obtained using primary antibodies conjugated with Alexa Fluor 546 and Alexa Fluor 488, respectively, at different scanning layers [(**A**), near the cell surface and (**B**), deeper in the cell towards the middle of its thickness]. Phalloidin-CF 405 for F-actin cytoskeleton and Hoechst 33342 for nuclei appear in surrogate grey; (**C**,**D**) immunofluorescence images of TRPM7 (stained in green when using Alexa Fluor 488) and F-actin cytoskeleton (stained in red when using Phalloidin-Alexa Fluor 546) were used for calculating fluorescence intensity in human (**C**) and pig (**D**) RA cardiomyocytes presented in (**E**,**F**). Hoechst 33342 for nuclei appears in blue. Scale bars indicate 20 µm; (**E**,**F**) constructed graphs of the fluorescence intensity using stacks of 12 slices for the localization of TRPM7 (green) and F-actin (red) along lines of interest on the cardiomyocytes merged images. The sampling distance in depth between scanned slices was 1 µm. The calculated correlation coefficient between F-actin and TRPM7 peaks (at a place as indicated) was 0.32 for human (**E**) and 0.11 for pig (**F**) RA cardiomyocytes.

As a first step, we verified the specificity of antibodies tested in support of the evidence in our previous study [3] showing that both TRPM6 and TRPM7 channels were indeed expressed in pig cardiomyocytes. In this previous study, the specificity of the immunostaining was examined using negative controls in which the primary antibodies were omitted. In the present study, the primary antibodies against these proteins were used but blocking peptides against these antibodies were included to serve as negative controls. It has been shown that such an approach provides higher specificity [21]. Figure 1A,B illustrate the immunofluorescence of TRPM6 and TRPM7 detected in pig RA cardiomyocytes incubated only with the primary antibodies. In contrast, Figure 1C,D show that no immunofluorescence was detected if the anti-TRPM6 or anti-TRPM7 antibodies were exposed to their respective blocking peptides. The antibodies displayed practically no cross-reactivity (off-target binding) with the blocking peptides. Figure 1E,F show that the immunofluorescence level obtained either when using the TRPM7 blocking peptide with anti-TRPM6 antibodies (0.03 ± 0.001 a.u.; *n* = 7) or the TRPM6 blocking peptide with anti-TRPM7 antibodies (0.11 ± 0.001 a.u.; *n* = 6) was similar to that obtained in the absence of the blocking peptides (0.03 ± 0.002 a.u. for TRPM6, *n* = 6, and 0.12 ± 0.002 a.u. for TRPM7, *n* = 10). Overall, these data point to a target-selective binding of both anti-TRPM6 and anti-TRPM7 antibodies. Qualitatively, the fluorescence spots of TRPM7 and TRPM6 both appeared to fall nearly systematically on the clear (less dark) zones of the cytoskeleton staining (see merged images in the lower panels of Figure 1A,B and also Figure 2A,B), resulting in transversal alignments.

**Figure 3:**
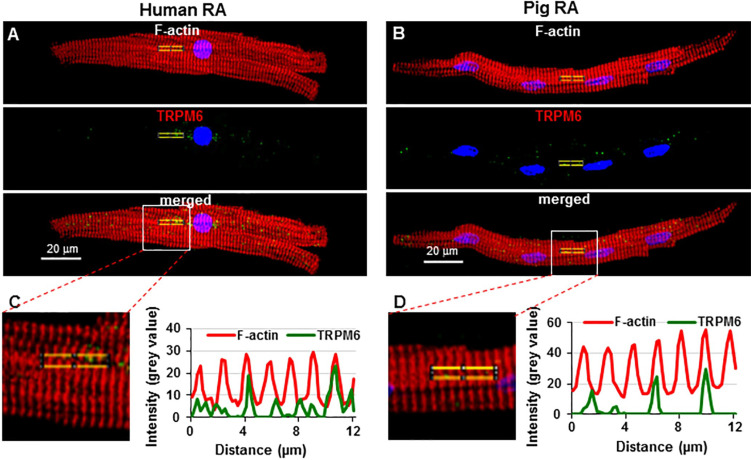
Cellular localization of TRPM6: (**A**,**B**) The immunofluorescence images of TRPM6 (stained in green when using Alexa Fluor 488) and F-actin cytoskeleton (stained in red when using Phalloidin-Alexa Fluor 546) were used for calculating fluorescence intensity in human (**A**) and porcine (**B**) RA cardiomyocytes presented in (**C**,**D**). Hoechst 33342 for nuclei appears in blue. Scale bars indicate 20 µm; (**C**,**D**) constructed graphs of the fluorescence intensity for the localization of TRPM6 (green) and F-actin (red) along lines of interest on the merged images of cardiomyocytes as indicated by the zoomed-in area. The calculated correlation coefficient between F-actin and TRPM6 peaks (at a place as indicated) was 0.25 for human (**C**) and 0.33 for pig (**D**) RA cardiomyocytes.

Similar results with the use of blocking peptides were obtained in human RA cardio- myocytes (not illustrated). Figure 2A,B illustrate the immunofluorescence of both TRPM6 and TRPM7 in the same human RA cardiomyocyte. There was also a correlation between TRPM fluorescence and cytoskeletal organization in human as well as pig cardiomyocytes (Figure 2E,F and Figure 3C,D). To quantitatively demonstrate the colocalization of TRPM6 or TRPM7 immunofluorescence with cellular cytoskeletal elements (stained in red), a scanning of the fluorescence intensity and of the light intensity due to striations was made along a small line segment of interest parallel to the major length of the cell. Figure 2E,F and Figure 3C,D show that TRPM7 or TRPM6 fluorescence peaks coincided with cytoskeletal fluorescence peaks. TRPM6 peaks were lower/less than those of TRPM7, but the same correlation remained apparent.

### 2.2. Sensitivity to Pharmacological Modulation

**Figure 4:**
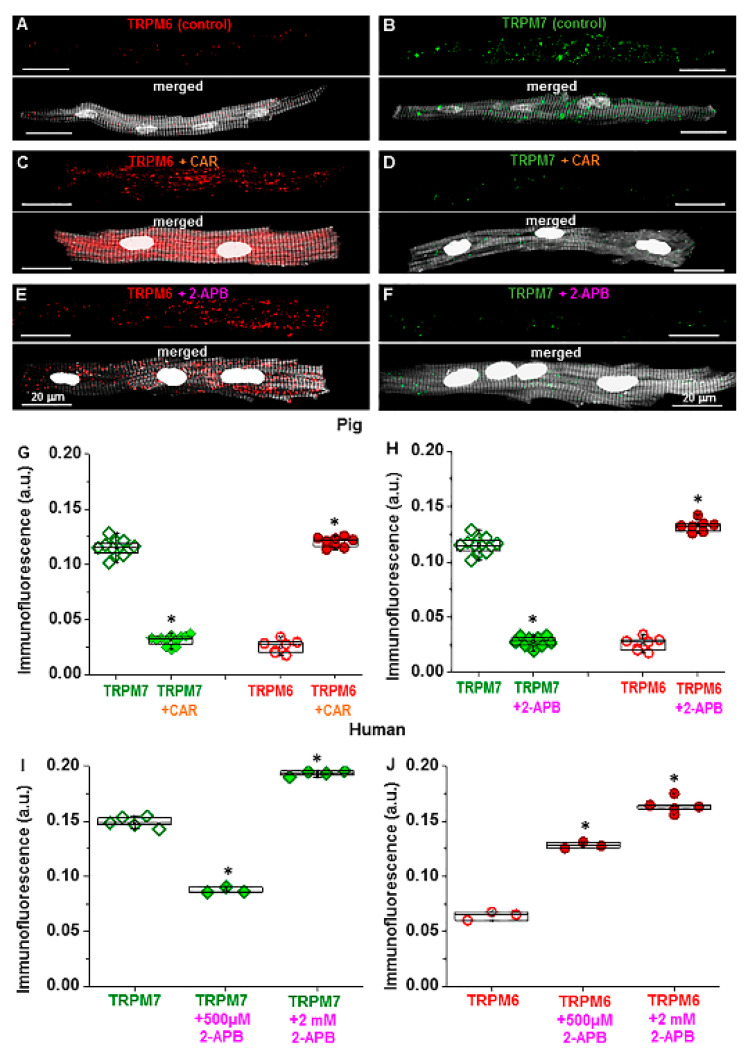
Effect of TRP channel modulating drugs carvacrol (CAR) or 2-aminoethoxydiphenyl borate (2-APB) on detected TRPM6 and TRPM7 in pig and human RA cardiomyocytes: (**A**–**F**) Immunofluorescence of TRPM6 and TPRM7 in pig cardiomyocytes under control conditions and in the presence of CAR or 2-APB. Scale bars indicate 20 µm; (**G**,**H**) quantification of the immunodetected TRPM7 and TRPM6 proteins in pig RA cardiomyocytes in the absence (unfilled symbols) or in the presence (filled symbols) of either CAR (**G**) or 2-APB (**H**); (**I**,**J**) quantification of the immunodetected TRPM7 (**I**) and TRPM6 (**J**) proteins in human RA cardiomyocytes in the absence (unfilled symbols) or presence (filled symbols) of 2-APB at either 500 µM or 2 mM. Fluorescence intensity expressed in arbitrary units (a.u.). * *p* < 0.001 for drug vs. no drug. Notice opposite changes on TRPM7 with 2-APB at 500 µM vs. at 2 mM.

Next, we tested the sensitivity of pig atrial cardiomyocytes to TRP channel modulators, given that our previous study demonstrated an alteration of TRPM channel detection in human cardiomyocytes [20]. Figure 4A–F show that incubating pig cells with the TRP channel modulators carvacrol (CAR) or 2-aminoethoxydiphenyl borate (2-APB) reduced TRPM7 immunofluorescence but increased that of TRPM6. As summarized in Figure 4G,H, in cells incubated for 15 min with CAR (100 µM), TRPM7 fluorescence was decreased from 0.12 ± 0.002 a.u. to 0.03 ± 0.001 a.u. (*n* = 10–12, *p* < 0.001). In contrast, TRPM6 fluorescence increased from 0.03 ± 0.002 a.u. to 0.13 ± 0.002 a.u. (*n* = 7 each, *p* < 0.001). Similar changes were caused by 2-APB (500 µM): decreased TRPM7 fluorescence from 0.12 ± 0.002 a.u. to 0.03 ± 0.001 a.u. (*n* = 12 each, *p* < 0.001), but increased TRPM6 fluorescence from 0.03 ± 0.002 a.u. to 0.12 ± 0.002 a.u. (*n* = 7–8, *p* < 0.001). Consistent with our previous findings [20], similar changes were obtained on TRPM7 and TRPM6 fluorescence in human atrial myocytes (Figure 4I,J).

Given the dose-dependent inhibitory vs. stimulatory effect of 2-APB on TRPM7 ion currents (measured electrophysiologically) in CHOCK1 cells [22], we also investigated the effect of 15-min preincubation with 2 mM 2-APB on human atrial cardiomyocytes. Figure 4I,J illustrate that, for TRPM7, instead of the decrease obtained with 2-APB at 500 μM, an increase was obtained at 2 mM. With 500 µM 2-APB immunodetected TRPM7 decreased from 0.15 ± 0.002 a.u. to 0.09 ± 0.002 a.u. (*n* = 3–5, *p* < 0.001), but with 2 mM the immunodetected TRPM7 increased to 0.20 ± 0.002 a.u. (*n* = 4, *p* < 0.001). For immunodetected TRPM6 measured under the same experimental conditions, the increase obtained at 500 µM (from 0.06 ± 0.002 a.u to 0.13 ± 0.002 a.u., *n* = 3, *p* < 0.001) was further enhanced at 2 mM (increase to 0.16 ± 0.003 a.u., *n* = 5, *p* < 0.001 vs. value in the absence of 2-APB), respectively. In pig atrial cardiomyocytes (not illustrated), similar to in human cells, the immunofluorescence for TRPM7 after 15-min preincubation with 2 mM 2-APB was increased (0.14 ± 0.005 a.u., *n* = 7 vs. 0.12 ± 0.002 a.u. in control, *n* = 20) in contrast to the decrease (0.03 ± 0.001 a.u., *n* = 10) obtained at 500 µM.

### 2.3. TRP Detection in Cardiac Tissue and Subcellular Localization

**Figure 5:**
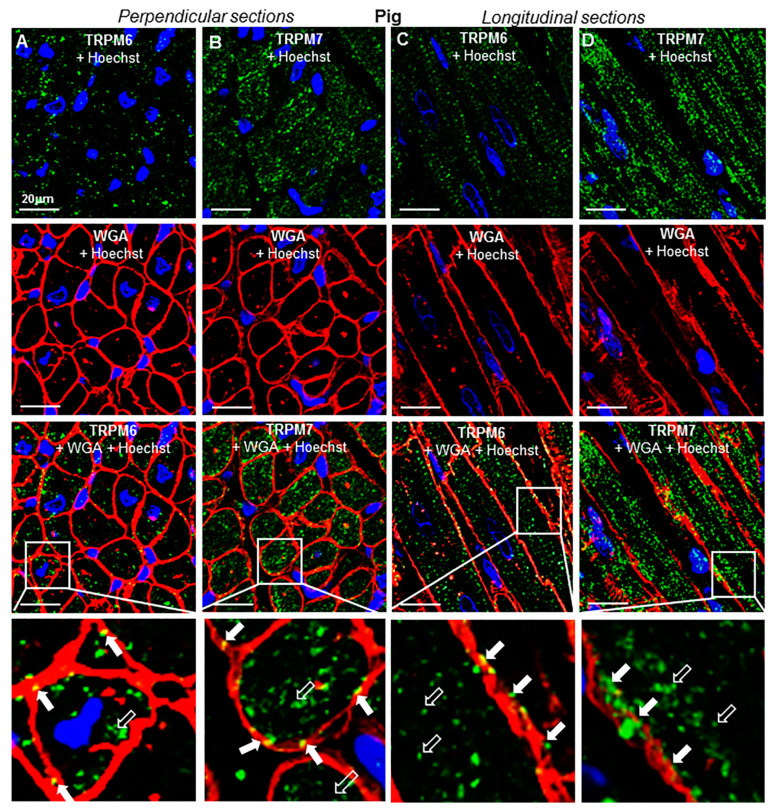
Tissue-level and subcellular localization of TRPM6 and TRPM7 by immunohistochemistry in the pig RA: (**A**–**D**) Confocal microscopy images obtained from perpendicular (**A**,**B**) and longitudinal (**C**,**D**) tissue slices. Images shown at higher magnification in the lower panels correspond to zoomed-in areas delimited by squares at a lower scale in the upper panels. TRPM6 and TRPM7 proteins were detected with AF-488-conjugated antibodies (green), the cell surface was labeled with AF-555 for wheat germ agglutinin (WGA, in red), whereas the nucleus staining (with Hoechst 33342) appears in blue. Notice that TRPM6 and TRPM7 are detected both at the cell membrane (filled arrows) and intracellularly (unfilled arrows). Scale bars indicate 20 µm.

**Figure 6:**
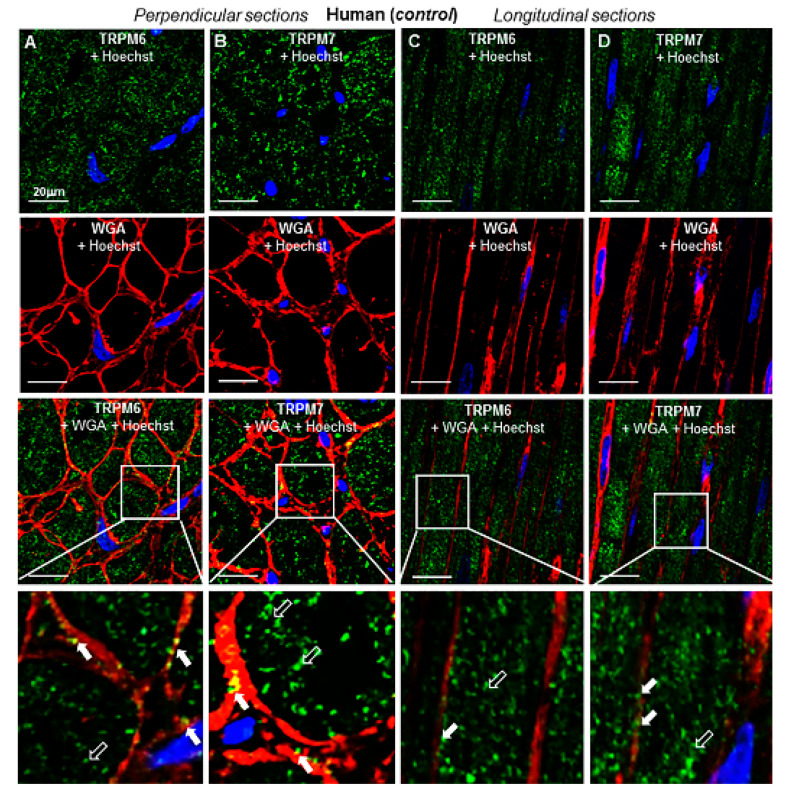
Tissue-level and subcellular localization of TRPM6 and TRPM7 by immunohistochemistry in the RA of an undiseased human heart from car victim. (**A**–**D**) Confocal microscopy images obtained from perpendicular (**A**,**B**) and longitudinal (**C**,**D**) tissue slices. Images shown at higher magnification in the lower panels correspond to zoomed-in areas delimited by squares at a lower scale in the upper panels. TRPM6 and TRPM7 proteins were detected with AF-488-conjugated antibodies (green), the cell surface was labeled with AF-555 for wheat germ agglutinin (WGA, in red), whereas the nucleus staining (with Hoechst 33342) appears in blue. Notice that TRPM6 and TRPM7 are detected both at the cell membrane (filled arrows) and intracellularly (unfilled arrows). Scale bars indicate 20 µm.

**Figure 7:**
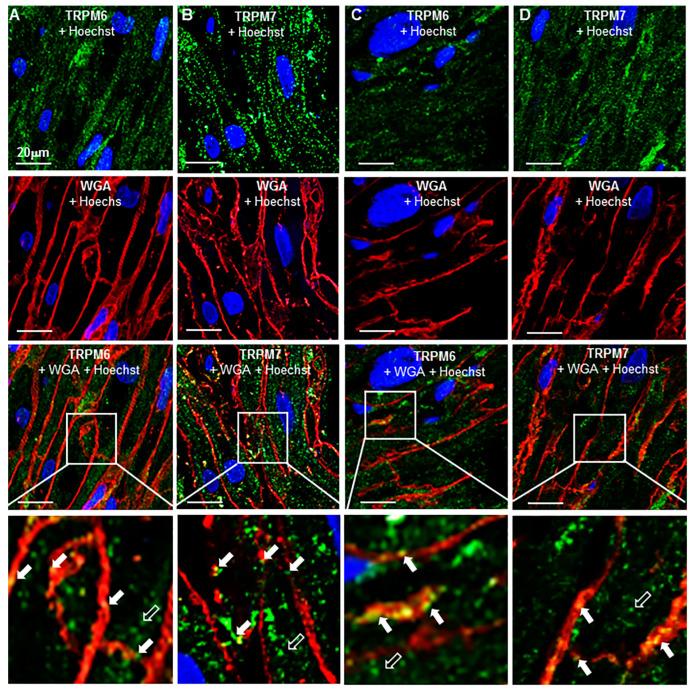
TRPM6 and TRPM7 detection by immunohistochemistry in diseased human RA tissue: (**A**–**D**) Representative images obtained from longitudinal slices of RA tissues from two different patients: an ischemic heart disease (IHD) patient (**A**,**B**) and an AF patient (**C**,**D**). The area labeled with the square bars corresponds to the images presented on a larger scale in the panels below them. AF-488 for the TRPM6 and TRPM7 protein appears in green. AF-555 for the cell membrane surface labeling with WGA appear in red, and Hoechst 33342 for the nucleus staining appear in blue. Arrows highlight the presence of TRP proteins. Notice, qualitatively, the amorphous distribution of TRPs and less colocalization with the cell membrane in both the IHD (**A**,**B**) and AF (**C**,**D**) hearts as compared with the control (car victim’s heart in Figure 6).

To evaluate the 3D spatial distribution of TRPs, immunohistochemical labeling was performed in transversal and longitudinal RA tissue sections. Figure 5 shows representative histotopograms of TRPM6 and TRPM7 immunofluorescence images in RA transversal (Figure 5A,B) and longitudinal (Figure 5C,D) tissue slices of the pig heart, whereas Figure 6 shows similar labelings in RA tissue slices of an undiseased human heart. The fluorescence of TRPM6 and TRPM7 was detected in both perpendicular and longitudinal slices. With the aid of cell surface labeling (using WGA), TRPM fluorescence was observed in the intracellular compartment as well as on or near the cell surface of transversal (see arrows) and longitudinal tissue slices (see also Figure 7), though qualitatively, the surface staining appeared less than the intracellular staining. There was a noticeable pattern in the disposition of the TRPM fluorescence spots, which on longitudinal images appeared to follow different lines along the major cell length. There was apparently no similar linear or other specific (e.g., radial) pattern on transversal slices.

### 2.4. Change of Expression by Diseases

Figure 7 illustrates TRPM6 and TRPM7 detection in longitudinal sections of atrial tissues from patients with ischemic heart disease (IHD, Figure 7A,B) or with AF (Figure 7C,D), respectively. Qualitatively, as compared with undiseased tissue (Figure 6), the fluorescence spots of TRPM7 and TRPM6 in diseased cardiac tissue both appeared to be less organized. Linear disposition of fluorescence spots along the cell length on longitudinal sections was present but less evident than in undiseased hearts, and the lines did not appear to fill the whole cell thickness. In addition, the TRPM fluorescence spots were less colocalized with the cell surface.

**Figure 8:**
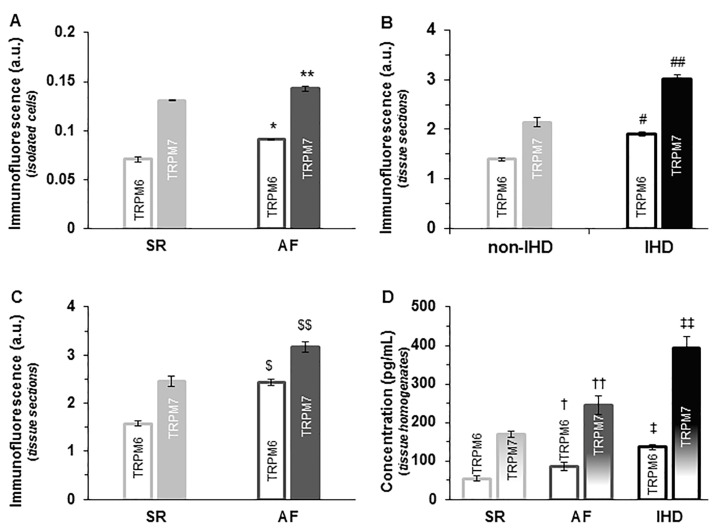
Effect of cardiac disease conditions on TRPM6 and TRPM7: (**A**–**C**) TRPM fluorescence intensity levels in RA cells (**A**) or tissues (**B**,**C**). Fluorescence intensity levels of TRPM6 (unfilled) and TRPM7 (filled) channels in isolated cardiomyocytes of the human heart, grouped according to the heart rhythm (SR vs. AF, (**A**)) or according to the absence or presence of ischemic heart disease (non-IHD vs. IHD, (**B**)). Fluorescence intensity levels of TRPM6 (unfilled) and TRPM7 (filled) channels in histological slices grouped according to the heart rhythm (SR vs. AF, (**C**)); (**D**) TRPM7 and TRPM6 protein levels in human heart tissue homogenates grouped according to the heart rhythm and the presence of ischemic disease. Mean data of immunofluorescence provided in arbitrary units (a.u) for TRPM6 and TRPM7: * and ** *p* < 0.05 AF vs. SR, respectively, in isolated cells; # and ## *p* < 0.01 IHD vs. non-IHD, respectively, in tissue sections; $ and $$ *p* < 0.01 AF vs. SR, respectively, in tissue sections, and mean data of TRPM6 and TRPM7 protein concentrations in pg/mL, † and †† *p* < 0.05 AF vs. SR, ‡ and ‡‡ *p* < 0.05 IHD vs. SR (non-IHD), respectively.

Figure 8 summarizes the fluorescence levels of TRPM6 and TRPM7 in the human RA tissues obtained in different disease groups. In general, the immunofluorescence levels of TRPM6 and TRPM7 proteins in atrial cells and tissues of AF hearts were both significantly (*p* < 0.05) higher than those in hearts with sinus rhythm (SR, Figure 8A,C). In isolated cardiomyocytes, the fluorescence intensity levels for TRPM6 and for TRPM7 for hearts in SR were 0.07 ± 0.002 a.u. and 0.13 ± 0.000 a.u., respectively (*n* = 14–16), whereas for hearts with AF the fluorescence levels were 0.09 ± 0.001 a.u. and 0.14 ± 0.002 a.u., respectively (*n* = 12–17, Figure 8A). In contrast to the data in isolated cardiac myocytes (where the fluorescence for TRPM7 is nearly one order of magnitude larger than for TRPM6), in cardiac tissue immunohistochemical analysis, the detected fluorescence intensities for TRPM6 and for TRPM7 were of the same order of magnitude, but still showed increased fluorescence levels (2.43 ± 0.067 a.u. and 3.16 ± 0.108 a.u., respectively, (*n* = 11–19) in AF vs. 1.58 ± 0.059 a.u. and 2.47 ± 0.109 a.u., respectively, (*n* = 11–19, *p* < 0.01) in SR) (Figure 8C). Furthermore, in IHD hearts, the fluorescence intensity levels of both TRPM6 and TRPM7 were also enhanced in immunohistochemical analysis as follows: 1.40 ± 0.034 a.u. for TRPM6 and 2.14 ± 0.088 a.u. for TRPM7 in non-IHD vs. 1.91 ± 0.042 a.u. for TRPM6 and 3.01 ± 0.091 a.u. for TRPM6 in IHD (*n* = 15 each, *p* < 0.01, Figure 8B).

The increased expression of TRPM6 and TRPM7 proteins in disease conditions was confirmed by an enzyme-linked immunosorbent assay (ELISA) as shown in Figure 8D. The detected TRPM6 and TRPM7 protein concentrations for human tissue homogenates in SR (non-IHD) were 53.1 ± 6.64 pg/mL and 169.4 ± 8.45 pg/mL, respectively (*n* = 11); for human tissue homogenates with AF, they were 84.8 ± 11.29 pg/mL and 245.1 ± 24.07 pg/mL, respectively (*n* = 9; *p* < 0.05 vs. SR); for human tissue homogenates with IHD, they were 135.9 ± 7.18 pg/mL and 392.6 ± 29.77 pg/mL, respectively (*n* = 12).

## 3. Discussion

In the present study, we demonstrate the expression of TRPM6 and TRPM7 in RA cardiomyocytes and tissues of the pig and human hearts. In confirmation of our previous data in the human heart [20], the immunolabeling with anti-TRPM6 or anti-TRPM7 antibodies appeared to be specific, since no immunofluorescence signal was detected upon blocking the test proteins with antibody-specific peptides. Thus, the present results strengthen the evidence that, apart from the ubiquitous TRPM7 [16,17], TRPM6 is also present in cardiac tissue (see also [15]). That said, it is important to note that the use of blocking peptides or the lack of cross-reactivity of TRPM6 and TRPM7 antibodies, demonstrated in the present study, does not represent absolute validation of antibody specificity. Nonetheless, in our preliminary studies (see Methods), we tested independent antibodies that were able to bind to different regions of the protein, but recognized the same target, and we showed that the antibodies were all able to detect TRPM6 or TRPM7. However, in pig, similar to observations in human cells, the TRPM6 signal was generally weaker than that of TRPM7, a feature that may partly explain why the expression of TRPM6 could have been missed in some previous cardiac studies. Nonetheless, in the present study, the two TRPMs could be distinguished by their different drug sensitivity profiles, since either CAR or 2-APB increased the expression of TRPM6 but suppressed that of TRPM7. Consistent with the expression profile observed in the present study, in other non-cardiac cell lines, CAR or 2-APB have also been shown to inhibit TRPM7 currents (measured by electrophysiological techniques) [23,24], whereas 2-APB activated TRPM6 currents [22]. Furthermore, in the present study, a higher (2 mM) 2-APB concentration enhanced (instead of further decreasing) TRPM7 fluorescence, consistent with the activation of TRPM7 currents also observed in CHOCK1 cells at high 2-APB concentrations [22]. Taken together, these results suggest that, although TRPM6 and TRPM7 are both expressed in cardiac tissue, these proteins are distinct and likely to be regulated differentially. However, this differential regulation does not exclude the formation of TRPM6-TRPM7 heteromers. It would be of interest to test whether the newly discovered TRPM6- vs. TRPM7-selective pharmacological tools [25] would be able to distinguish ion current components due to either protein in electrophysiological experiments on cardiac cells.

Presently, it remains difficult to account for the rapid changes in fluorescence signals induced by the inhibitors, especially for 2-APB with opposite effects observed at low vs. high drug concentrations. The magnitude of the fluorescence changes are too large to be easily accounted for by a mechanism involving protein expression. The fact that the drug-induced changes in electrical signals also show concentration-dependent opposite effects for TRPM7 [22], as is also the case for TRPM2 [26], suggests that complex mechanisms are implicated in the observed drug action. We could not find any previous study that reported 2-APB- or CAR-induced changes of protein detection or expression. Based on the fact that we detected proteins localized at the cell surface and intracellularly (see below), the drug-induced changes are not attributable to changes in cell membrane insertion of presynthesized vesicles. It is possible that the drugs induce conformational changes that alter detection by antibodies. As far as other 2-APB- and/or CAR-sensitive TRP channels are concerned, it is known that either drug binds to domains in the channel protein, causing conformational changes that modify the interaction of the channels with surrounding substances or structures. For those channels, the stimulatory or inhibitory actions of 2-APB or CAR are proposed to be due to positive or negative allosteric effects [27,28]. To the best of our knowledge, binding sites for 2-APB or CAR in TRPM6 or TRPM7 channels have not been defined. Conceivably, such an allosteric modulation of TRPM channel proteins could also result in a modification of the affinity or the efficacy to interact with the antibodies used for protein detection and account for the modified fluorescence. The involvement of multiple binding sites within a protein could then account for opposite effects of a given drug at low vs. at high concentrations. The experiments of the present study did not address this possibility, which requires further investigation.

The present study also addressed issues of the subcellular localization of the TRPM channels and the relation with cytoskeletal proteins. In their subcellular distribution, TRPM6 and TRPM7 proteins were localized both intracellularly and on/near the cell surface, albeit with the surface staining being far less than the intracellular staining. The cell surface TRPM localization is consistent with the channels’ activity measurable by electrophysiological techniques, but the implication of intracellular localization is not clear. It is possible that newly synthesized TRPM proteins that have not yet been inserted into surface membranes could also be detectable intracellularly. In addition, though we could not ascertain whether the intracellular staining was on intracellular membrane structures, part of the intracellular TRPM protein pool could be associated with intracellular membranes or vesicles, since the presence/role of TRPM proteins in intracellular vesicular membranes has been reported (e.g., see [29,30]). In addition to the presence in surface or intracellular vesicular membranes, the channel proteins are likely present at the level of contractile proteins. TRPM6 and TRPM7 proteins showed both a transverse and longitudinal alignment with the axis of the cardiomyocyte. The TRPM6 and TRPM7 immunofluorescence spots were located in the clear (less dark) bands of the cells and the peaks of TRPM signals corresponded to those of the F-actin cytoskeleton staining. If the less dark zones represent the I bands of the sarcomere, then the coincidence of the TRPM spots with these zones is probably an indication that the proteins are present at the Z line (which is normally in the middle of the I band), and that they colocalize with Z line proteins (actinin or other). The alignment with the longitudinal axis is probably related to the cytoskeleton proteins within successive Z lines in a given microfibril along the longitudinal axis [31], seen as longitudinal bright spots in cytoskeleton images independently of the TRPM labeling (seen in lower panels of Figure 1A,B, Figure 2A,B and Figure 4A–F).

The functional role of cardiac TRPM channels has remained unknown. The fact that most immunofluorescence spots are intracellular implies that TRPM channel activity, measured electrophysiologically (i.e., located in the cell membrane), is not representative of all channels detected by immunolabeling. The proximity or association of TRPM6 and TRPM7 with contractile cytoskeletal structure is suggestive of a role of these proteins in the regulation of cardiac cell contraction. In non-muscle cells, TRPM7 is known to interact with myosin and may regulate actomyosin function either directly by phosphorylating the myosin heavy chain [32] or indirectly by ERK-mediated phosphorylation of the myosin light chains [33]. This regulation of myosin is mainly due to the kinase function of the TRPM proteins and results in regulation of polarized cell movement and of podocyte formation [32,34]. It is not known whether such interactions can also occur with cardiac myosin. Except for an association of TRPM7 overexpression with diminished cardiac contractile function following cobalt intoxication [35], to the best of our knowledge, no study, as yet, has identified an influence of TRPM6 or TRPM7 on cardiac mechanical function. Despite the proximity with F-actin, TRPM proteins did not mediate any phosphorylation of actin [32].

In the present study, we show the upregulated expression of TRPM6 and TRPM7 in AF and IHD, as indicated by the higher fluorescence intensities and homogenate protein concentrations. In addition, there was a disruption in spatial distribution of the TRPMs in disease, as indicated by the less organized appearance of intracellular fluorescence spots and less colocalization with surface membrane structures. Since structural remodeling with loss and disruption of contractile proteins exists in disease conditions such as AF [36,37] and chronic ischemia [38], the loss of linear alignment of TRPM6 and TRPM7 immunofluorescence spots might be related to the relative disorganization of myofilaments. It should be noted that a fluorescence intensity analysis of TRPM6 and TRPM7 by immunohistochemistry in tissues is likely to be less informative than immunostaining in cardiomyocytes (see Figure 8). This is due to the morphological structure of the heart that includes contamination by interstitial fibrosis etc., which could be changed during the development and the aggravation of heart failure (see Figure 7). Nonetheless, our findings support the data showing that underlying diseases influence the levels of immunodetected TRPM6 and TRPM7 proteins [20], but the mechanisms of action still require further studies. TRPM7 has also been shown to be upregulated (at mRNA level) in end-stage heart failure [39]. In addition, when expressed in cardiac fibroblasts, TRPM7 has profibrotic effects in cardiac disease conditions such as myocardial infarction [40], AF [8], and sick sinus syndrome [41].

In conclusion, our study demonstrates that TRPM6 and TRPM7 proteins can be detected in cardiac atrial tissue and that the TRPMs have relatively similar subcellular localization, but different drug sensitivity profiles. This study also demonstrates an upregulated expression of TRPM6 and TRPM7 in IHD and AF, which suggests a possible role of the channels in the pathophysiology of cardiac atrial disease.

## 4. Materials and Methods

### 4.1. Acquisition of Cardiac Samples

Atrial samples from hearts of healthy pigs and of either diseased (i.e., IHD and AF) or undiseased human subjects were used in this study. This study was carried out in accordance with the European Community guiding principles outlined in the Declaration of Helsinki and the *Guide for the Care and Use of Laboratory Animals*. Experiments on pigs were approved by the State Food and Veterinary Service of the Republic of Lithuania (No. G2-68, 21 June 2017). The experiments on human samples were approved by the Ethics Committee of Biomedical Research of Kaunas Region, Lithuania (No. BE-2-71, 22 December 2017). Human cardiac samples were obtained as biopsy specimens during open heart surgery, or as tissue resected from explanted failing hearts, or resected postmortem from undiseased (unused transplantation donor) hearts of car accident victims. The overall characteristics of the open heart surgery patients are summarized in Table 1. Written and informed consent was obtained. Pathohistological diagnosis was made in the Department of Pathology, Lithuanian University of Health Sciences.

The samples were used for detection of TRPM7 and TRPM6 proteins either with immunofluorescence on enzymatically dissociated cardiomyocytes or on atrial tissue slices. The concentrations of the TRPM proteins were also measured with ELISA.

### 4.2. Immunofluorescence

Cardiomyocytes for immunofluorescence were enzymatic dissociated from small tissue chunks, as previously described [3,5,20]. The cells were permeabilized and incubated with primary polyclonal anti-TRPM7 antibodies (#ACC-047; Alomone Labs, Israel; dilution 1:200, targeting the CKRRKKDKTSDGPKLFLTEE peptide, corresponding to amino acid residues 1146–1165 of human TRPM7) or rabbit polyclonal anti-TRPM6 antibodies (#ACC-046 (Alomone Labs, Jerusalem, Israel) dilution 1:200, targeting the CVKDYDLERGPDEK peptide, corresponding to amino acid residues 802–815 of TRPM6). Of note, on the pig preparations, we applied the same polyclonal antibodies that react with human epitopes [20]. Although the results from only a few antibody types are reported in the present study, in our preliminary studies we tested antibodies obtained from various companies, antibodies conjugated or not with Alexa Fluor, and antibodies which recognize different amino acid (a.a.) sequences. For the recognition of TRPM7 we tested antibodies from: Thermo Fisher (S74-25) (MA5-27620), which recognize a.a. sequence 1817–1863 (C-terminus of mouse); Alomone (ACC-047), which recognize a.a. sequence 1146–1165 (C-terminus of human); Abcam (ab 85016), which recognize a.a. sequence 1817–1863 (cytoplasmic C-terminus of mouse); Bioss Antibodies (bs-9044R-A488, conjugated with AF488), which recognize a.a. sequence 801–900/1865; and Novus Bio (NBP1-20224) which recognize 4th cytoplasmic loop of human. For the recognition of TRPM6, we used antibodies from: Alomone (ACC-046), which recognize a.a. sequence 802–815 of mouse extracellular loop; and Santa-Cruz (sc-365536, conjugated with AF546), which recognize internal region of human. In some of the samples, antibody-blocking peptides (obtained from Alomone Labs, Jerusalem, Israel) were applied by mixing part of the prepared stock solution containing the primary antibodies with the peptides, according to the manufacturer’s instructions. The blocking peptide for anti-TRPM7 antibody (#BLP-CC047) and the blocking peptide for anti-TRPM6 (#BPL-CC046) was created by Alomone Labs for the antibody TRPM6 or TRPM7.

Thereafter, cells were incubated for 1.5 h with a fluorescent-labeled secondary antibody (either donkey anti-rabbit IgG Alexa Fluor 488 conjugate A21206 (Invitrogen, Thermo Fisher Scientific, Rockford, IL, USA) dilution 1:200 or goat anti-rabbit IgG Alexa Fluor 546 conjugate A11035, Invitrogen (Thermo Fisher Scientific, Rockford, IL, USA) dilution 1:200), co-stained (for 30 min) with Phalloidin-Alexa Fluor 546 (A22283, Invitrogen (Thermo Fisher Scientific, Waltham, MA, USA) dilution 1:100) or with Phalloidin-CF 405 (00034, Biotium, Fremont, CA, USA) dilution 1:100) and with Hoechst 33342 (B2261 (Sigma-Aldrich, St. Louis, MO, USA) 25 µg/mL, for 10 min) for labeling of the F-actin cytoskeleton and of the nucleus, respectively. We also used mouse monoclonal anti-TRPM6 conjugated with AF546 (sc-365536, Santa Cruz Biotechnology, Inc., Dallas, TX, USA) dilution 1:50 and rabbit polyclonal anti-TRPM7 conjugate with AF-488 (bs-9044R-A488, Bioss Antibodies, Woburn, MA, USA) dilution 1:200. Glass slides with stained cells were covered with ProLong Gold Anti-fade reagent (P36934, Molecular Probes, Thermo Fisher Scientific, Waltham, MA, USA) and coverslip glass, and sealed with clear nail polish. Cardiomyocytes were visualized under a confocal laser scanning microscope (Olympus FV1000, Olympus Corporation, Tokyo, Japan) magnification ×60, from which images were taken using the same scanning parameters for both at the TRPM7 and TRPM6 proteins. Images are presented as stacks of 10–12 slices at fixed intensity. Cardiomyocyte area (pixels) and fluorescence intensity were measured in stacks using the ImageJ (NIH, USA) and Imaris (Bitplane AG, Zurich, Switzerland) softwares. Immunodetected TRPM6 and TRPM7 protein levels were calculated using the formula: fluorescence intensity * 1000/cell area. In order to reduce any statistical confounder, immunofluorescence reading was blinded, as the conditions used to keep cells were unknown to the person performing the reading.

In the present study, for immunofluorescence of cardiomyocytes on atrial tissue slices, longitudinal and transversal slices were obtained from the same tissues. Specimens of formalin-fixed and paraffin-embedded heart muscle tissues were sliced (3 μm thick), placed onto Super Frost Plus slides (Menzel, Germany), and sections were deparaffinized. The epitope retrieval procedure was performed using a microwave tissue processor RHS-1 (Milestone Medical, Bergamo, Italy), incubating samples in TRIS/EDTA buffer (pH 9.0) at 110 °C temperature for 8 min. The sections were incubated for 3 h with primary rabbit polyclonal anti-TRPM7 antibody conjugated with Alexa Fluor 488 (NBP1-20224, Novus Biologicals, Centennial, CO, USA) dilution 1:200 or primary mouse monoclonal anti-TRPM6 antibody conjugated with Alexa Fluor 488, 1:50 dilution (sc-365536, Santa Cruz Biotechnology, Inc., USA). In cardiac myocytes, partial localization of detected proteins in the cell surface was aided by labeling with wheat germ agglutinin (WGA, which labels both the cellular membranes and the capillaries [42] and could be stained (in red) using Alexa Fluor 555). The nucleus was labeled (for 15 min) with Hoechst 33342, 1 μg/mL (B2261, Sigma-Aldrich, USA). For the reduction of autofluorescence, the tissue slices were incubated with the Vector TrueVIEW (Vector Laboratories), and thereafter, were covered with antifade mounting medium VECTASHIELD Vibrance (Vector Laboratories). The histopathological evaluation was performed under a confocal laser scanning microscope Olympus FV1000 (Olympus Corporation, Japan). Immunofluorescence reading was blinded, as the conditions used to prepare atrial tissue slices were unknown to the person performing the reading.

### 4.3. ELISA Assay

ELISA kits (MyBioSource, Inc., San Diego, CA, USA) were used to quantitatively determine concentrations of TRPM6 and TRPM7 proteins in human tissue homogenates. Optical signals were recorded with a spectrometer (Multiskan MF, Thermo Fisher Scientific, Finland) at a wavelength of approximately 450 nm. The levels of TRPM6 and TRPM7 were determined according to instructions provided by the ELISA kits for TRPM6 (MBS457214) and TRPM7 (MBS457216). After measuring the optical densities of the standard solutions, a standard curve was drawn using the Curve Expert 1.4 software (Daniel G. Hyams, Hyams Development, Chattanooga, TN, USA). Then, the concentrations of TRPM6 and TRPM7 in the tissue homogenates were determined by comparing the optical density of the samples to the standard curve.

### 4.4. Chemicals

2-APB was obtained from Abcam (ab120124, Cambridge, UK) and carvacrol (CAR, ≥97% purity) was purchased from Carl Roth GmbH + Co (Karlsruhe, Germany), whereas the other chemicals were from Sigma Aldrich (St. Louis, MO, USA). The highest concentration of the solvent (DMSO) was <0.1% and did not affect the measurements.

### 4.5. Data Analysis

Average data are presented as mean ± standard error of the mean (SEM). Means were compared using the two-tailed unpaired *t*-test or ANOVA for evaluating differences between groups; *p* < 0.05 was taken as threshold for statistical significance.

## Figures and Tables

**Table 1:** Clinical characteristics of the patients.

Patient Data	SR	IHD	AF
Age range (years)	29–79	54–87	40–80
Mean age (years) ± SEM	53.0 ± 11.76	65.9 ± 9.59	63.2 ± 11.11
Female, *n* (%)	3 (17.7)	8 (26.7)	6 (35.3)
Male, *n* (%)	14 (82.4)	22 (73.3)	11 (64.7)
Total, *n* (%)	17 (100)	30 (100)	17 (100)
**Surgical intervention**			
Valve surgery, *n* (%)	12 (70.6)	0 (0)	13 (76.5)
CABG surgery, *n* (%)	0 (0)	14 (46.7)	2 (11.8)
CABG and Valve surgery, *n* (%)	0 (0)	11 (36.7)	1 (5.9)
Transplantation, *n* (%)	5 (29.4)	5 (16.7)	1 (5.9)

AF: atrial fibrillation; CABG: coronary artery bypass graft; IHD: ischemic heart disease.

## Data Availability

Data are available upon request.

## Abbreviations

2-APB, 2-aminoethoxydiphenyl borate; AF, atrial fibrillation; CAR, carvacrol; ELISA, enzyme-linked immunosorbent assay; IHD, ischemic heart disease; RA, right atrium; SR, sinus rhythm; TRPM, transient receptor potential melastatin (TRPM) ion channels.
